# Using topic modeling via non-negative matrix factorization to identify relationships between genetic variants and disease phenotypes: A case study of Lipoprotein(a) (*LPA*)

**DOI:** 10.1371/journal.pone.0212112

**Published:** 2019-02-13

**Authors:** Juan Zhao, QiPing Feng, Patrick Wu, Jeremy L. Warner, Joshua C. Denny, Wei-Qi Wei

**Affiliations:** 1 Department of Biomedical Informatics, Vanderbilt University Medical Center, Nashville, TN, United States of America; 2 Division of Clinical Pharmacology, Vanderbilt University Medical Center, Nashville, TN, United States of America; 3 Medical Scientist Training Program, Vanderbilt University School of Medicine, Nashville, TN, United States of America; 4 Department of Medicine, Vanderbilt University Medical Center, Nashville, TN, United States of America; New Jersey Institute of Technology, UNITED STATES

## Abstract

Genome-wide and phenome-wide association studies are commonly used to identify important relationships between genetic variants and phenotypes. Most studies have treated diseases as independent variables and suffered from the burden of multiple adjustment due to the large number of genetic variants and disease phenotypes. In this study, we used topic modeling via non-negative matrix factorization (NMF) for identifying associations between disease phenotypes and genetic variants. Topic modeling is an unsupervised machine learning approach that can be used to learn patterns from electronic health record data. We chose the single nucleotide polymorphism (SNP) rs10455872 in *LPA* as the predictor since it has been shown to be associated with increased risk of hyperlipidemia and cardiovascular diseases (CVD). Using data of 12,759 individuals with electronic health records (EHR) and linked DNA samples at Vanderbilt University Medical Center, we trained a topic model using NMF from 1,853 distinct phenotypes and identified six topics. We tested their associations with rs10455872 in *LPA*. Topics enriched for CVD and hyperlipidemia had positive correlations with rs10455872 *(P* < 0.001), replicating a previous finding. We also identified a negative correlation between *LPA* and a topic enriched for lung cancer (*P* < 0.001) which was not previously identified via phenome-wide scanning. We were able to replicate the top finding in a separate dataset. Our results demonstrate the applicability of topic modeling in exploring the relationship between genetic variants and clinical diseases.

Using topic modeling via non-negative matrix factorization to identify relationships between genetic variants and disease phenotypes: A case study of Lipoprotein(a) (*LPA*)

## Introduction

Elucidating associations between genetic variants and human diseases creates new avenues for disease prevention and enables precise identification and treatment of diseases [1,2]. During the past two decades, genetic studies have uncovered thousands of genetic variants that influence risk for disease phenotypes [3], e.g., the discovery of a variant in proprotein convertase subtilisin/kexin type 9 (*PCSK9[4]*) associated with low plasma low-density lipoprotein, which led to a new therapeutic drug class that was approved by the US Food and Drug Administration in 2015. Many of these discoveries come from large-scale association analyses. The two most notable approaches are genome-wide (GWAS) and phenome-wide association studies (PheWAS) [2, 5]. For a given phenotype, GWAS scans hundreds of thousands to millions of single nucleotide polymorphisms (SNPs) across the genome in a hypothesis-free approach. PheWAS, conversely, analyzes thousands of disease phenotypes compared to a single SNP. In a GWAS, the outcome variable is a disease phenotype and predictor variables are SNPs. In a PheWAS, the outcome variable is a SNP and predictor variables are disease phenotypes. Although the output is different, these techniques share many commonalities.

In particular, association analyses test many predictors at one time and assume that each predictor has an independent effect. Nevertheless, diseases often occur as a group of comorbidities, e.g. hyperlipidemia (HLD) and cardiovascular diseases (CVDs). Conventional association analyses may not capture the inter-connections among variables such as phenotypes and thus may not be sensitive to identify important genotype-phenotype relationships. Moreover, association analyses also face the challenge of scaling to an increasing number of phenotypes. Previously, we have described a “networked PheWAS” approach which can address interconnectivity but still requires a degree of supervised interpretation [6].

This study tested the feasibility of topic modeling, an unsupervised machine learning method, for identifying relationships between genetic variants and disease phenotypes. Topic modeling was initially introduced as a text mining technique [7,8]. It extracts latent topics or themes from documents and thus facilitates the understanding of data [9,10]. Each document can have multiple topics. Each topic can be represented by related words (or other inputs). Topic modeling has achieved notable applications in text mining [11,12], social networks [13] and computer vision [14]. Recently, it has been brought into the biomedical research field, primarily focusing on clinical text mining [15–17]. A few studies applied this technique to mining EHRs’ events [17–21] and capturing their relationship with the genetic information [15,22,23], e.g. McCoy et al. used latent Dirichlet allocation to find disease clusters and examined their associations with the polygenic risk scores of depression [20].

Our study utilized non-negative matrix factorization (NMF), a topic modeling method to one of the largest biobanks in the U.S. We hypothesized that topic modeling could identify disease clusters among the phenome, which can help replicate known findings and uncover unidentified relationships between genetic variants and disease phenotypes. We used NMF [24,25] to identify latent topics (e.g. disease clusters or relevant comorbidities) from EHR data. We then tested associations between the EHR-derived topics and a lipoprotein(a) (*LPA*) SNP (rs10455872). We chose this *LPA* SNP because previous studies have shown that high levels of the *LPA* protein product, Lp(a), are associated with increased risks of developing HLD and CVD [26]. Specifically, the *LPA* SNP (rs10455872), as a single variant, explains 20–30% of the variation in circulating Lp(a) levels, which makes it an ideal candidate for this study [27]. To demonstrate the benefit of using topic modeling, we compared our result with a traditional PheWAS approach.

## Materials and methods

### Study cohort

We used data from BioVU, the de-identified DNA biobank at Vanderbilt University Medical Center (VUMC), to conduct this study. BioVU contains DNA samples from >250,000 individuals linked with de-identified EHRs, including diagnostic and procedure codes, clinical notes, laboratory values and medications. We limited our study within a group of selected individuals who received regular, longitudinal care at Vanderbilt to avoid the incomplete data issue. We identified 12,759 adult individuals of European ancestry (Female/Male: 6,018/6,741; mean age: 70.3±12.3) who had both EHRs and genotyped data of rs10455872.

### rs10455872 Genotyping

We extracted individual’s rs10455872 information from available genotype data. All genotyping was previously conducted using commercially available genome-wide SNP arrays with quality control criteria for variants. Genotype imputation was conducted on the Michigan Imputation server[28] with minimac3[29], using the Haplotype Reference Consortium reference panel, version r1.1[30].

Among the cohort of 12,759 individuals, we observed 85.2% AA, 14.2% AG, 0.6% GG. The minor allele frequency (MAF) of the rs10455872 G allele is 7.7% in our cohort, consistent with the 7% MAF in the European population [31]. We used 0, 1, and 2 to represent the number of *LPA* rs10455872 G alleles that each individual carries.

### Disease phenotypes

Following established protocols used in past studies [32], we grouped each individual’s International Classification of Disease, 9^th^ edition (ICD-9-CM) codes into disease “phecodes” [33]. There were 1853 phecodes extracted from 12,759 individuals. For each phecode, we labeled individuals with the phecode with a ‘1’, and those having no such phecode with a ‘0’.

### Topic modeling via non-negative matrix factorization (NMF)

**Fig 1:**
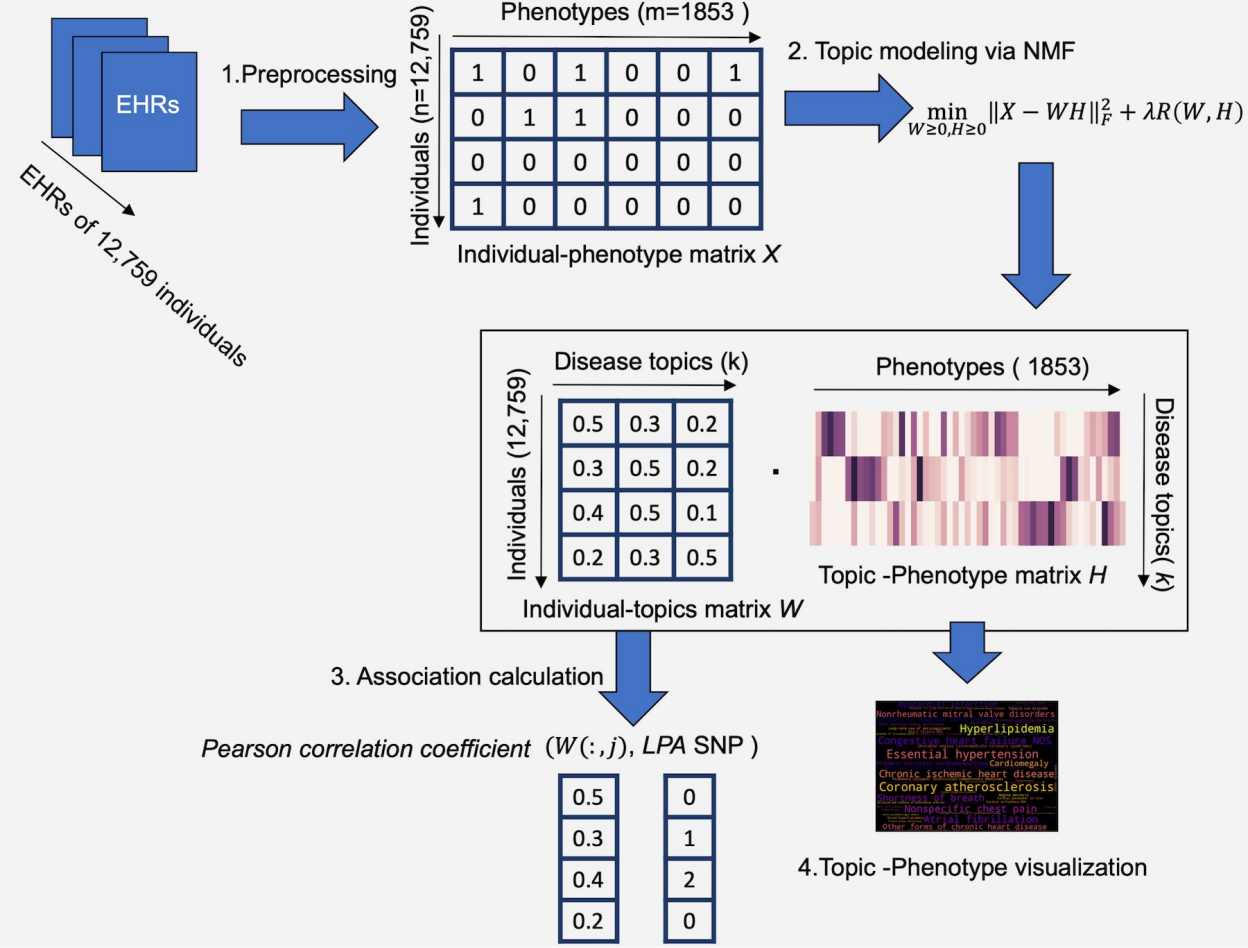
Illustration of topic modeling on EHRs using NMF.

We use topic modeling via NMF to extract a set of topics (i.e. clusters of disease) from individuals’ phenotypes data (Fig 1). In this study, we used a data matrix *X* of dimensions *n*×*m* to represent the input data, where *n* denotes the number of an individual (e.g. *n* = 12,759), and *m* denotes the size of the phecodes (e.g. *m* = 1853). The entry of the matrix *X*_*ij*_∈*X* was a binary value (0 or 1) indicating whether *i*^th^ individual had the *j*^th^ phecode.

NMF is based on assumption that a *n× m* sparse data matrix *X* of with *n* samples and *m* dimensions, which can often be represented by a small sets of *k* basis vectors. The linear combination is a *n× k* coefficients matrix *W*, which is a lower-dimensional representation for *X*.

To be more specific, the input data matrix *X* can be approximately represented by the product of two non-negative matrices *W* and *H*, such that

$$\min_{W\geq 0,H\geq 0}{\left\|X-WH\right\|}_{F}^{2}+\lambda R(W,H),$$ (1)

where ${\left\|X-WH\right\|}_{F}^{2}$ is a Frobenius norm to measure the error between approximation and the original data, that is, ${\left\|X-WH\right\|}_{F}^{2}=\sum_{i,j}{\left(X-WH\right)}_{ij}^{2}.\;\lambda R(W,H)$ is a regularization term, where *λ* is the parameters of the regularization term.

*H* is a *k*×*m* topic–phenotype matrix with *k* rows and *m* columns, where *k* denotes the size of topics and *m* is the size of phenotypes. It is useful to view each row vector in *H* as a disease topic represented by a set of phenotypes, where each cell value defines the phenotype’ rank in the topic.

*W* is an *n*×*k* individual-topic matrix, with *n* individuals and *k* topics. We can view the row vector of *W* as an individual’s EHR document with a cell value indicating the individual’s relevance to a disease topic. *W* is then used for the association analysis between the topics and rs10455872.

*R*(*W*, *H*) is the regularization term that combines *L*_*1*_ and *L*_*2*_ norms, which is defined as:

$$R(W,H)=\gamma ({\left\|W\right\|}_{1}+{\left\|H\right\|}_{1})+\frac{1}{2}(1-\gamma )({\left\|W\right\|}_{F}^{2}+{\left\|H\right\|}_{F}^{2}),$$ (2)

where γ is the ratio for *L*_*1*_ penalty. The regularization term is not necessarily included in NMF, but adding the regularization term may help balance the sparsity of the topics, as we assume that each individual may have a small set of diseases.

### Topic evaluation

To evaluate topics results, we asked domain experts (authors with clinical background) to view the words describing a topic to determinate if the topic is semantically meaningful. In addition, we used two objective measures, which were also commonly used in topic modeling evaluation—topic dependency and topic coherence (S1 Text) in [34,35]. Topic dependency reflects the overlapping between topics by calculating mean pairwise Jaccard similarity between the topic descriptors (i.e. top-ranked words in a topic). A higher number of similar terms between topics suggest that topics are overlapped (less useful) [34]. Topic coherence reflects if a topic can represent a single theme or similar concepts by measuring the co-concurrency of the topic descriptors in the whole documents. Topics with higher topic coherence are usually easy to interpret.

Since pre-set values of parameters such as γ and *λ* may impact the results, we tuned the parameters and used agreement scores to measure the stabilities of topics. We calculate the stability of topics using Jaccard similarity and Hungarian method [36]. A higher agreement score indicates a higher stability, i.e., the concepts in a topic remain consistent regardless of the parameter values.

To visualize the topic results, we employed word clouds to present top-ranked phenotypes in a topic. We used 2-dimensional (2D) visualizations to visualize the disease clusters of the cohort by using t-Distributed Stochastic Neighbor Embedding (t-SNE) [37].

### Statistical analysis

We tested the association between each extracted topic and *LPA* SNP variation by applying Pearson correlation coefficient (PCC) and logistic regression (LR). PCC measures the strength of a linear association between two variables, and generates a correlation coefficient denoted by *r*∈[−1,1], showing correlation direction. To compute PCC, we used each topic vector of individual-topic matrix *W* in NMF as the predictor variable *x*, where each cell defines the relevance scores on each individual. We used the vector of *LPA* SNP, where each cell represents the number of risk allele, as the variable *y*. For LR, we used the topic vector as the predictor, and *LPA* SNP as the outcome variable (counts of alleles > 1 is treated as 1), adjusting for age and sex (i.e. 1 represents female; 0 represents male). We reported the coefficient and *p*-value for each predictor.

To compare the results, we also conducted a conventional PheWAS in our cohort (i.e. 12,759 individuals) to test the association between for *LPA* SNP with 1853 phenotypes. PheWAS is a systematic approach to replicate and discover relationships between targeted genotypes and multiple phenotypes [5].

### Validation Cohort

To demonstrate the generalizability of this approach, we also repeated the process using the data from a separate cohort and tested whether or not we can replicate our findings. The data was collected from another project to study stains. All individuals are European ancestry and under statin treatment. The cohort had 3889 adult individuals with more percentage of males and similar age as the study cohort (Male/Female:2,473/1,416 [63.6% vs 52.8%]; mean age: 71.9±11.3 [71.9 vs 70.3]). The study cohort and the validation cohort were mutually exclusive.

## Results

**Fig 2:**
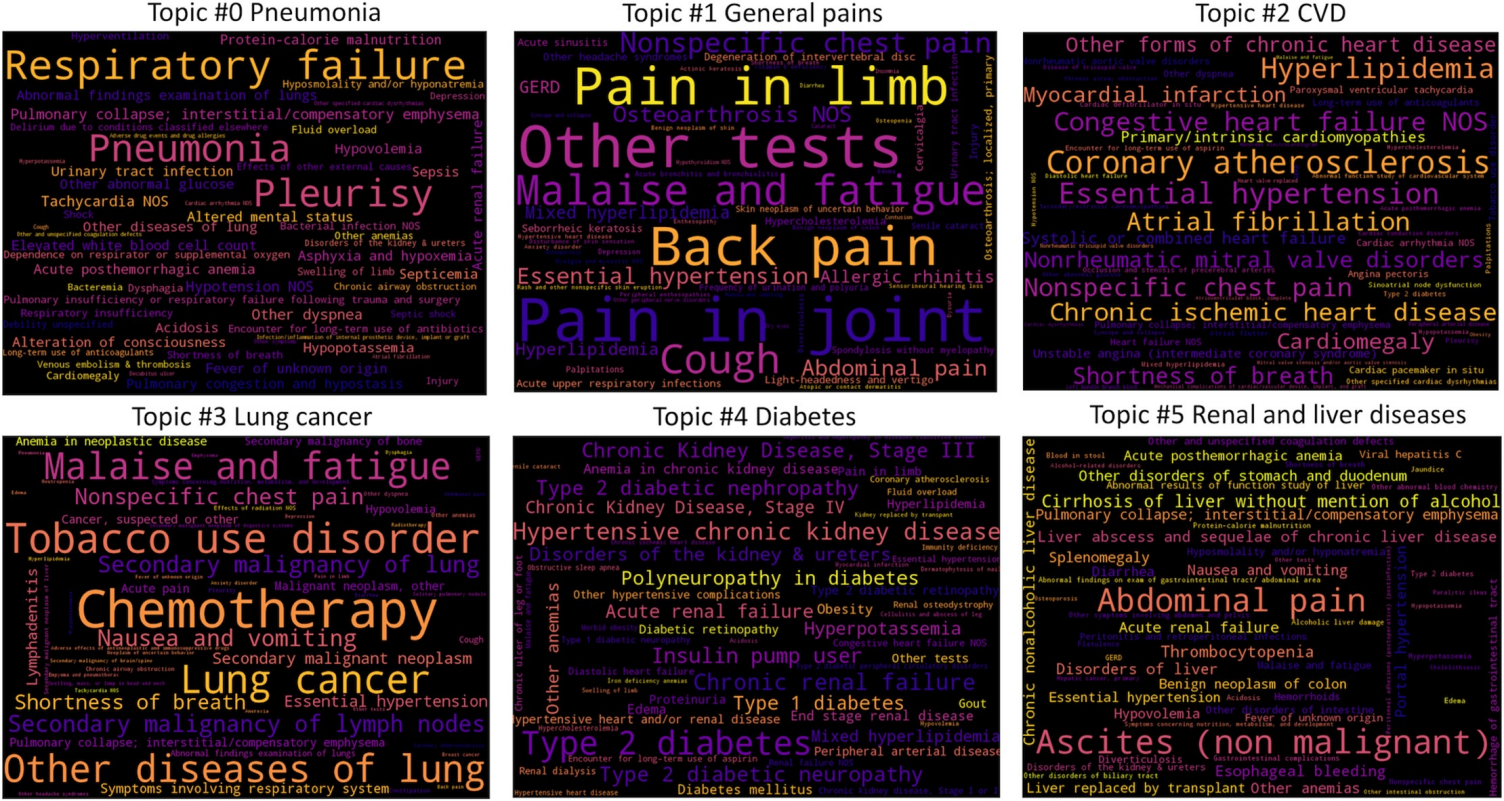
Word clouds for six topics. The size of the words (phecode) in each cloud indicates the weights of the phenotypes on the topic. Phenotypes with larger-sized words have greater influence on the topic compared to phenotypes with smaller-sized words. For each word cloud, we listed the top 60 words.

We applied a topic modeling algorithm using NMF on the dataset of 12,759 individuals and obtained six topics. Topics are reviewed by a clinician to ensure clinically meaningful, and labeled with a major disease (Fig 2). The topics were coherent and consistent with the comorbidities associated with the phenotypes that is most prevalent in the cohort. For example, topic #2 defined diseases related to CVD (e.g., HLD, hypertension, and chronic ischemic heart disease), topic #3 represented phenotypes relevant to lung cancer and its treatment.

**Fig 3:**
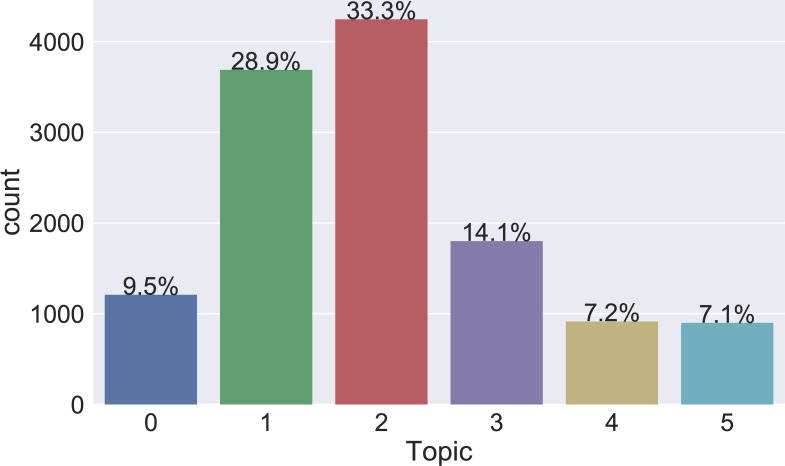
Topic distribution in the cohort. To visualize the prevalence of each topic in the cohort, we assigned an individual to the topic with the maximum score.

We plot the distribution of the numbers of topics in the cohort in Fig 3. Topic #2 was the most prevalent (33%) topic in the cohort. Topics #1 and #3 were the second and third most prevalent topics in the cohort. We also plotted the scores of individual-phenotypes matrix (*W)* with boxplot in S1 Fig.

**Fig 4:**
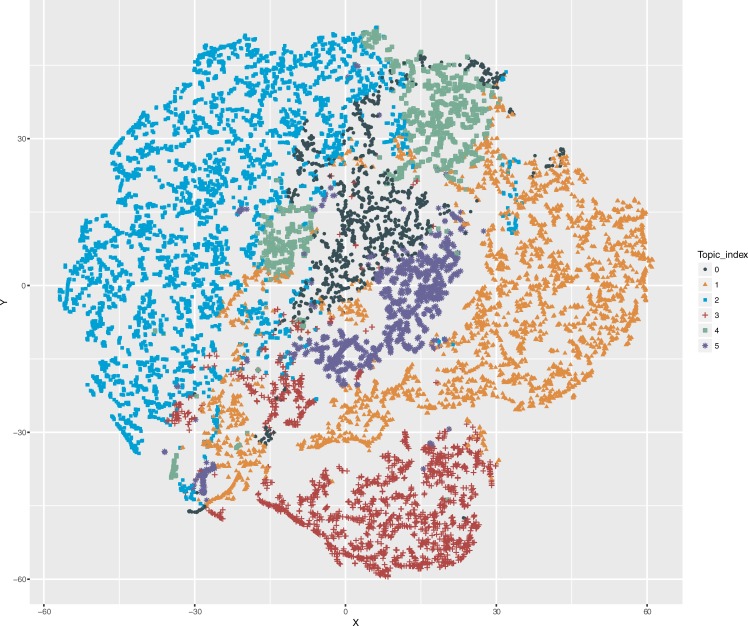
t-SNE plot of visualizing the patient clusters in a projected 2D metric map (The perplexity was set to 30).

We present the visualization of topic modeling results by t-SNE in Fig 4. Each data point represented an individual. We labeled each individual with the assigned topic. We used principal component analysis (PCA) for t-SNE embedding initialization. PCA is a feature reduction method to project high-dimensional data into a lower dimensional space that can explain the most variance of the input data. PCA initialization is more globally stable than random initialization[38]. Consistent with our clinical observation, the topic #2 contains the largest number of individuals in the cohort.

The PCC association test suggests that topic #2 and #3 were significantly associated with rs10455872 (Table 1). Topic #2, a group of lipid and cardiovascular diseases, had a weak but significant positive correlation with rs10455872 (*r* = 0.072, *p* = 5.8e-16). We also found that topic #3, a group of phenotypes relevant to lung cancer, had a weak but significant negative correlation with rs10455872 (*r* = -0.039, *p* = 8.5e-6). Although the *r* coefficient is weaker than the topic#2, these correlations are highly statistically significant.

**Table 1:** Pearson correlation coefficient testing between LPA variant for each topic.

Topic	Top phenotypes in this topic	*r*	*P*-value
#0	Respiratory failure, Pneumonia, Pleurisy, Pulmonary collapse; interstitial/compensatory emphysema, Hypotension NOS, Tachycardia NOS, Other dyspnea, Hypopotassemia, Sepsis, Septicemia	0.011	0.199
#1	Pain in joint, Other tests, Back pain, Pain in limb, Malaise and fatigue, Cough, Nonspecific chest pain, Essential hypertension, Osteoarthrosis NOS, Abdominal pain	-0.008	0.358
#2	Coronary atherosclerosis, Essential hypertension, Hyperlipidemia, Congestive heart failure NOS, Nonspecific chest pain, Atrial fibrillation, Chronic ischemic heart disease, Shortness of breath, Nonrheumatic mitral valve disorders, Cardiomegaly	0.072	5.8e-16
#3	Chemotherapy, Tobacco use disorder, Lung cancer, Other diseases of lung, Malaise and fatigue, Secondary malignancy of lymph nodes, Secondary malignancy of lung, Nausea and vomiting, Nonspecific chest pain, Shortness of breath	-0.039	8.5e-6
#4	Type 2 diabetes, Hypertensive chronic kidney disease, Chronic renal failure, Insulin pump user, Type 2 diabetic neuropathy, Chronic Kidney Disease, Stage III, Type 2 diabetic nephropathy, Type 1 diabetes, Polyneuropathy in diabetes, Acute renal failure	0.002	0.783
#5	Ascites (nonmalignant), Abdominal pain, Cirrhosis of liver without mention of alcohol, Thrombocytopenia, Liver abscess and sequelae of chronic liver disease, Portal hypertension, Chronic nonalcoholic liver disease, Disorders of liver, Esophageal bleeding, Nausea and vomiting	-0.02	0.021

The LR analysis achieved a similar result in the direction and significance for most topics (Table 2). Particularly, topic #2 (CVD) had a positive correlation with rs10455872 (coefficient = 2.789, *p* = 3.42E-13), and topic #3 (lung cancer) vary inversely with rs10455872 (coefficient = -1.101, *p* = 0.009), which was consistent with PCC test.

**Table 2:** Logistic regression analysis between LPA variant for each topic.

Predictor	Coefficient	*P*-value
Age	-0.003	0.079
Sex	0.145	0.005
topic_0	0.542	0.166
topic_1	-0.07	0.820
topic_2	2.789	3.42E-13
topic_3	-1.101	0.009
topic_4	-0.446	0.275
topic_5	-0.695	0.131

**Fig 5:**
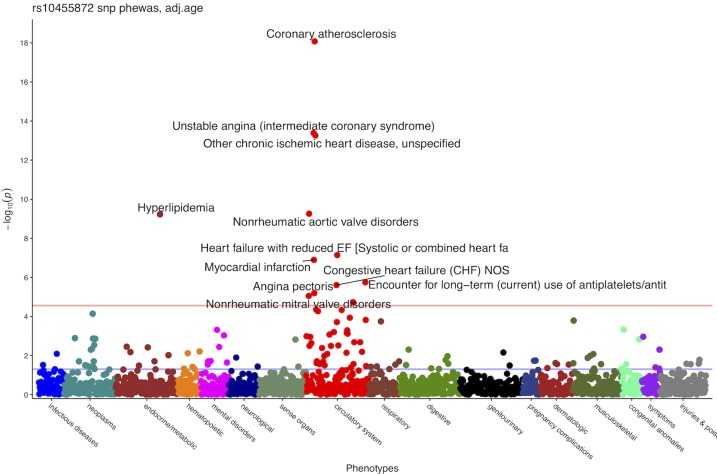
PheWAS results of rs10455872 on 12,759 individuals adjusted by sex and age.

PheWAS results on the same data (Fig 5) suggested a significant association between rs10455872 and phenotypes including coronary atherosclerosis, unstable angina, hyperlipidemia and myocardial infarction. Most of these phenotypes were present in topic #2. Phenotypes about neoplasms had the second higher associations. However, the association (p-value = 7.209713e-05) did not cross the Bonferroni.

We also validated the approach using data from a separate cohort, without any patient overlap with the study’s original cohort. The replicated cohort contains 3889 adults of European ancestry with a larger percentage of males (63.6% vs 52.8%) and similar age (71.9 vs 70.3). The results suggested one positively associated topic containing similar diseases including Coronary atherosclerosis, Essential hypertension, Hyperlipidemia, Nonspecific chest pain, Atrial fibrillation (S6 and S7 Figs, S1 and S2 Tables). We were not able to validate the lung cancer topic, which may due to the smaller data set and limited statistical power.

## Discussion

In this paper, we applied topic modeling to explore associations between disease phenotypes and genetic variants. We assumed that some disease phenotypes found simultaneously in EHR have correlated semantic meanings and thus can be learned as topics. We examined the associations between an *LPA* variant (rs10455872) and the six topics derived from EHRs. We observed the expected association between rs10455872 and a topic representing CVD/HLD. We also found a novel association, as of this writing [39], between the *LPA* variant and a lung cancer topic.

The *LPA* gene encodes lipoprotein (a), a major component of the Lp(a) particle. Individuals with elevated Lp(a) levels are more likely to develop CVD compared to those with normal or low Lp(a) level [27,40]. Approximately 70% of Lp(a) variation can be attributed to variants at the *LPA* locus [41–43], and rs10455872 alone explains ~25% variation in circulating Lp(a) levels [27]. Further, a previous genetic study suggested that *LPA* variants were strong predictors for CVD risk [27]. In a more recent study of >10,000 patients taking statins, we found that rs10455872 predicted residual CVD risk while on lipid-lowering treatment [44]. This study’s finding of a significant association between rs10455872 and the CVD/HLD topic demonstrates the feasibility of topic modeling as a critical tool for uncovering genotype-phenotype relationships.

We also observed a negative correlation between the *LPA* variant and the cancer/lung cancer topic, i.e., possessing this variant is protective. Previous epidemiological studies have reported that individuals with low Lp(a) levels had increased risk of all-cause and cancer-related mortality [45]. Mieno et al. found that hypolipoproteinemia(a) is a risk factor for cancer except for lung cancer. Nevertheless, there are few reports on a relationship between cancer and *LPA* polymorphism or expression levels. Our PheWAS analysis on the same cohort identified an association between rs10455872 and secondary malignancy of lymph nodes with borderline p-value (10e-5) but insignificance. To further explore this association between rs10455872 and the cancer/lung cancer topic, we queried gene2pheno (https://imlab.shinyapps.io/gene2pheno_ukb_neale/), which is a publicly available database for testing associations between predicted gene expression levels and phenotypes using data from the UK Biobank. Genetically predicted LPA expression levels were associated with death from T cell lymphomas (p = 6.9e-5, Underlying [primary] cause of death: ICD10: C84.5 Other and unspecified T-cell lymphomas). Given that lung cancer is strongly mediated by environmental exposure and that tobacco use disorder was also part of topic #4, it is possible that the SNP is a marker for propensity to smoking, e.g., similar to what was shown for rs16969968 [46]. Further genetic and epidemiological studies are needed to elucidate the relationship between Lp(a) levels and cancer incidence.

Compared with traditional PheWAS, we identified the most significant associations–between rs10455872 and CVD related diseases, consistent with PheWAS. We also identified a significant association between rs10455872 and cancer/lung cancer, which did not cross the Bonferroni in PheWAS. Besides, our approach automatically identified comorbidities and the interconnections between phenotypes, which cannot be easily identified by PheWAS. We conducted an association test of the comorbidities as a whole instead of each independent phenotype. For example, hyperlipidemia often presents with hypertension, as co-occurred in our topic #2. PheWAS can identify the significant association between the genetic variant and hyperlipidemia but cannot automatically identify the cluster of hyperlipidemias and test the relationship between the variant and the cluster.

Compared with existing work on using topic modeling on EHR events [19,20], we explored the choice of number of topics, evaluated the quality of the topics and tested on replicated cohorts.

### Choice of number of topics

Topic modeling approaches require pre-specification of the number of topics *k*. In this study, we set *k* = 6, because we focused on capturing the most prevalent diseases such as CVD for quantifying the association. Larger *k* may allow the discover associations between rare diseases and genetic variants, but increases the risk of fracturing common phenotype clusters.

It can be seen that (Fig 4), except for topic #4 (diabetes), the learned topics formed distinct clusters, indicating a good quality of topic modeling. Some of points in topic #4 (diabetes) were close with topic #2 (CVD), which was expected, because type II diabetes is an important risk factor that increases the risk of developing CVD. Compared to the other topics, #1 (Pain), #2 (CVD), and #3 (Lung Cancer) have more concentrated clusters.

For optimal selection of *k* , common approaches includes looking at the error in optimization or having domain experts review the topics to identify which set of topics are most meaningful, and have estimated *k* using singular value decomposition (SVD) to look at the decay of singular values [47–49]. We showed the decay of eigenvalues of the input data using the scree plot (S2 Fig). The top 5–6 components can explain the most variance of the input data. Therefore, we chose *k* = 6 in this study. Additionally, we also tried *k* = [10, 20,30]. Topics such as CVD and Lung cancer remained associated (S1 Appendix).

We evaluated the topic dependency and average topic coherence by varying *k* from 5 to 50 in S3 and S4 Figs [34]. The topic dependency drops when *k* increases from 5 to 15, and then became stable. The average coherences of topics kept dropping as *k* increases, which means that as the number of topics increases, it becomes difficult to interpret the meaning of the topics.

### Stability evaluation

We evaluated the impact of the parameters values (i.e. number of topics *k*, regularization parameters λ and γ in equations [2] and [3]) on the topic modeling results and the correlation study. In the study, we used *λ* = 0.2 and γ = 0.5 as the default setting. We compared the agreement scores of topics generated on different settings of *λ* and *r* with their default values. Results in S5 Fig indicated that *λ* and γ did not impact the consistency of the topic meaning. We also listed top-ranked phenotypes for each topic and statistical analysis results by using different combinations of *λ and* γ on *k* = [10, 20, 30] (S1 Appendix). CVD and lung cancer topics were present in all parameter’s settings, and their correlations with LPA from PCC and LR analysis were consistent with topics at k = 6 and the default values of *λ* and *r*.

### Limitations

There are several limitations in this study. First, we tested only one genetic variant in one gene. Rs10455872 explains approximate 25% change in circulating Lp(a) levels according to previous studies; however, it would be interesting to generate a genetic risk score for Lp(a) levels and test its association with disease phenotypes in the future. Second, we used a binary value to indicate if an individual had a diagnosis code. A method accounting for disease severity (e.g., counts of diagnosis codes) could be used in future studies. Finally, the current study was limited to using billing codes to phenotype individuals. We did not include other information, e.g. laboratory test and medications, to assign more accurate phenotypes. This problem can be solved in the future using more sophisticated “deep” phenotyping methods that include more features from EHRs.

### Conclusion

In summary, unlike traditional PheWAS that have treated each disease phenotype as a distinct variable, topic modeling via NMF generates more abstract latent factors from disease phenotypes and significantly reduces the number of multiple tests. Our results demonstrate the power of topic modeling in the detection of disease clusters and previously unexplored genotype-phenotype relationships among a large cohort.

## Supporting information

S1 Text Topic evaluation algorithms. (DOCX)

S1 Appendix Topic modeling and correlation study results with *topic k = 10*, *20*,*30 and various settings of λ, γ*. (XLSX)

S1 Fig Boxplot of individual-phenotypes matrix *W* (after *l2* normalization) to visualize the topic distribution in the cohort. (EPS)

S2 Fig Scree plot of topics for *k*∈[1, 20]. This scree plot shows that the eigenvalues start to form a straight line after the sixth principal component. Therefore, the remaining principal components account for a small proportion of the variability and may be less important.
(EPS)

S3 Fig Topic dependency measured by mean pairwise Jaccard similarity for different *k*. (EPS)

S4 Fig Topic coherence for different k. (EPS)

S5 Fig Topic stability with tuning parameters *λ*∈[0, 2] and *γ* = [0.5, 1] on *k* = [6, 10, 20, 30] A: Topic stability for varying λ∈[0, 2] on γ = 0.5; Topic stability for varying λ∈[0, 2] on γ = 1.
(EPS)

S6 Fig Scree plot of topics for *k*∈[1, 20] on replicated cohort. This scree plot shows that between the second and third eigenvalues, a straight line starts to form after the third principal component. Therefore, we chose to use 3 topics in the validation study, as the remaining principal components account for a small proportion of variability in the data, and thus may be less important.
(EPS)

S7 Fig Word clouds for three topics on replicated cohort. (TIFF)

S1 Table Pearson correlation coefficient testing between LPA variant for each topic on replicated cohort. * indicates significant association (p<0.05).
(DOCX)

S2 Table Logistic regression result between LPA variant for each topic on replicated cohort. * indicates significant association (p<0.05).
(DOCX)
