# Supplementary material for: Using topic modeling via non-negative matrix factorization to identify relationships between genetic variants and disease phenotypes: A case study of Lipoprotein(a) (LPA)
Source: PLoS One. 2019 Feb 13;14(2):e0212112. doi: 10.1371/journal.pone.0212112 (PMC6374022; doi:10.1371/journal.pone.0212112)
Supplement: S1 Text — (DOCX) [file pone.0212112.s001.docx]

**S1 Text Topic evaluation algorithms**

1. Algrothims1: Topic dependency calculation using mean pairwise Jaccard similarity

Let TD_i_ denotes the topic descriptors (e.g. top-ranked words) in a topic *i*.

The mean pairwise Jaccard similarity between the topic descriptors TD was calculated as follows:

$$MPJ= \frac{1}{\binom{k}{2}\sum_{j=2}^{k} \sum_{i=1}^{j-1} \frac{\left| {TD}_{i}\cap{TD}_{j} \right|}{\left| {TD}_{i}\cup{TD}_{j} \right|}}$$

, where *k* is the number of topics.

1. Algrothims2: Topic coherence calculation

Topic coherence is defined as follows:

Coherence (*V*) = $\sum_{(v_{i},v_{j} )\in V} score (v_{i},, v_{j},\epsilon)$,

where V is a set of word describing the topic and $\epsilon$is to ensure the scores return true values. We used $\epsilon$ =10e-12, in this study.

To calculate the score between each word in the topics, we used UMass metric.

$$score \left( v_{i},, v_{j},\epsilon\right)=log\frac{D\left( v_{i},v_{j} \right)+\epsilon}{D(v_{j})}$$

, where $D\left( v_{i},v_{j} \right)$ counts the number of documents containing words $v_{i}$ and $v_{j}$ and $D(v_{j})$counts the number of documents containing $v_{j}$.

1. Algrothims3: Topic agreement calculation.

The calculation is based on [25]. We used the code in https://github.com/derekgreene/topic-stability.
