# Supplementary figures and images for: Using topic modeling via non-negative matrix factorization to identify relationships between genetic variants and disease phenotypes: A case study of Lipoprotein(a) (LPA)
Source: PLoS One. 2019 Feb 13;14(2):e0212112. doi: 10.1371/journal.pone.0212112 (PMC6374022; doi:10.1371/journal.pone.0212112)

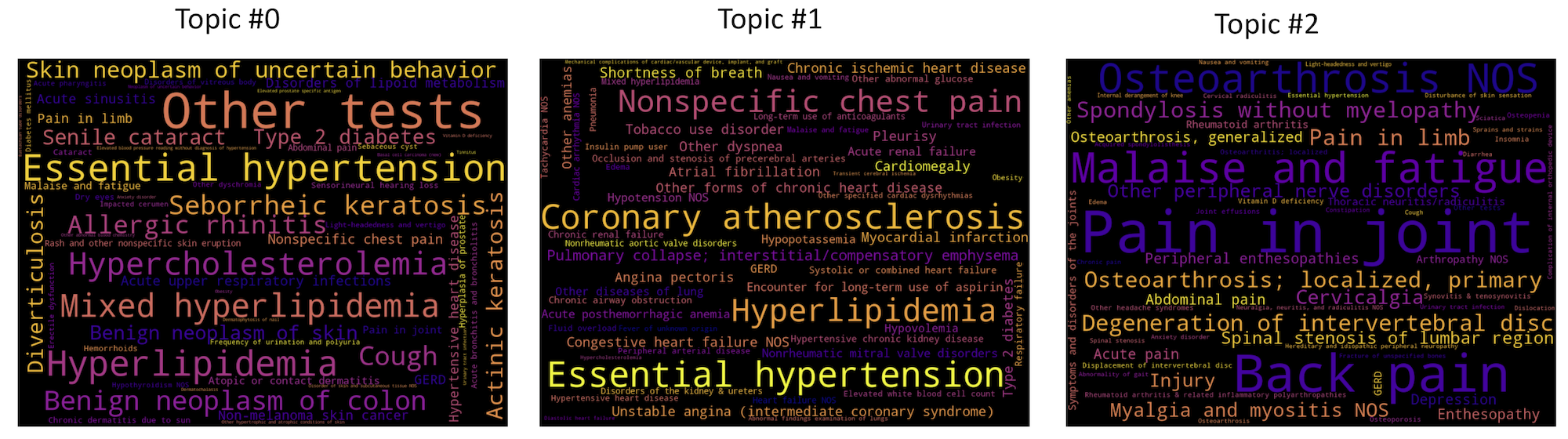

Supplement: S7 Fig — (TIFF) [file pone.0212112.s009.tiff]
