# Supplementary material for: Using topic modeling via non-negative matrix factorization to identify relationships between genetic variants and disease phenotypes: A case study of Lipoprotein(a) (LPA)
Source: PLoS One. 2019 Feb 13;14(2):e0212112. doi: 10.1371/journal.pone.0212112 (PMC6374022; doi:10.1371/journal.pone.0212112)
Supplement: S1 Table — * indicates significant association (p<0.05). (DOCX) [file pone.0212112.s010.docx]

**S1 Table.** Pearson correlation coefficient testing between LPA variant for each topic. * indicates significant association (p<0.05).

| **Topic** | **Top phenotypes in this topic** | ***r*** | ***P*-value** |
| --- | --- | --- | --- |
| Topic #0 | Other tests, Essential hypertension, Hyperlipidemia, Mixed hyperlipidemia, Hypercholesterolemia, Benign neoplasm of colon, Seborrheic keratosis, Cough, Allergic rhinitis, Skin neoplasm of uncertain behavior | -0.021 | 0.183 |
| Topic #1 | Essential hypertension, Coronary atherosclerosis, Hyperlipidemia, Nonspecific chest pain, Pulmonary collapse; interstitial/compensatory emphysema, Shortness of breath, Other dyspnea, Tobacco use disorder, Type 2 diabetes, Myocardial infarction | 0.053 | 9.00E-04* |
| Topic #2 | Pain in joint, Back pain, Malaise and fatigue, Osteoarthrosis NOS, Degeneration of intervertebral disc, Osteoarthrosis; localized, primary, Pain in limb, Spondylosis without myelopathy, Cervicalgia, Myalgia and myositis NOS | -0.023 | 0.150 |
