# Supplementary material for: Using topic modeling via non-negative matrix factorization to identify relationships between genetic variants and disease phenotypes: A case study of Lipoprotein(a) (LPA)
Source: PLoS One. 2019 Feb 13;14(2):e0212112. doi: 10.1371/journal.pone.0212112 (PMC6374022; doi:10.1371/journal.pone.0212112)
Supplement: S2 Table — * indicates significant association (p<0.05). (DOCX) [file pone.0212112.s011.docx]

**Table s2.** **Logistic regression result between LPA variant for each topic.** * indicates significant association (p<0.05).

| **Predictor** | **Coefficient** | ***P*-value** |
| --- | --- | --- |
| Age | -0.007 | 0.063 |
| Sex | 0.110 | 0.251 |
| topic_0 | -0.364 | 0.573 |
| topic_1 | 1.145 | 1.94E-03* |
| topic_2 | -0.968 | 0.132 |
